# Sex-specific trends in ventricular arrhythmias among European implantable cardioverter-defibrillator recipients

**DOI:** 10.1093/europace/euag268

**Published:** 2026-09-18

**Authors:** Andrea Papa, Sing-Chien Yap, Michael Kühne, Christian Sticherling, Felix Mahfoud, Dominic A M J Theuns, Beat Schaer

**Affiliations:** Clinic for Cardiology, University Hospital Basel, Petersgraben 4, Basel 4031, Switzerland; Cardiovascular Research Institute Basel, Spitalstrasse 2, 4056 Basel, Switzerland; Department of Cardiology, Erasmus MC, Rotterdam, The Netherlands; Clinic for Cardiology, University Hospital Basel, Petersgraben 4, Basel 4031, Switzerland; Cardiovascular Research Institute Basel, Spitalstrasse 2, 4056 Basel, Switzerland; Clinic for Cardiology, University Hospital Basel, Petersgraben 4, Basel 4031, Switzerland; Cardiovascular Research Institute Basel, Spitalstrasse 2, 4056 Basel, Switzerland; Clinic for Cardiology, University Hospital Basel, Petersgraben 4, Basel 4031, Switzerland; Cardiovascular Research Institute Basel, Spitalstrasse 2, 4056 Basel, Switzerland; Department of Cardiology, Erasmus MC, Rotterdam, The Netherlands; Clinic for Cardiology, University Hospital Basel, Petersgraben 4, Basel 4031, Switzerland; Cardiovascular Research Institute Basel, Spitalstrasse 2, 4056 Basel, Switzerland

## Introduction

Despite guideline-based indications, women are less frequently treated with implantable cardioverter-defibrillators (ICDs) than men.^1,2^ Prior studies reported fewer ventricular arrhythmias and appropriate ICD therapies in women, although with similar or even better survival^3–8^. However, whether sex-related differences have remained stable across ICD implantation periods in routine practice remains uncertain. We therefore examined sex-specific temporal trends in ventricular arrhythmias and mortality in a bi-centre ICD registry from Erasmus Medical Center, Rotterdam, and the University Hospital of Basel, between 2002 and 2017.

## Methods

We analysed consecutive adults (aged ≥18 years) undergoing *de novo* ICD implantations between 2002 and 2017, grouped into four implantation periods: 2002–2005, 2006–2009, 2010–2013, and 2014–2017. The overall cohort and temporal trends have been described previously.^9^ ICD indications were determined by treating teams according to contemporaneous guidelines. EPS was not routinely used for primary-prevention risk stratification and was not systematically recorded. The primary outcome was appropriate ICD therapy within 60 months after implantation. Additional outcomes included ventricular tachycardia, true ventricular fibrillation, and all-cause death. The proportional-hazards assumption was assessed using Schoenfeld residuals and formal residual–time testing and was violated for implantation-period effects. We therefore used multivariable logistic regression for the fixed 60-months endpoint. Prespecified covariates included sex, age, left ventricular ejection fraction (LVEF), QRS duration, ischaemic cardiomyopathy, atrial fibrillation at implant, diabetes, chronic kidney disease, prevention indication, use of CRT-D, and implantation period. Prespecified interaction terms were sex × time period, sex × CRT-D, and sex × QRS duration. Exploratory sex-stratified models used the same covariates.

## Results

Among 2184 patients, 300 (13.7%) were women. Women were younger at the time of implantation than men (63 ± 11 vs. 65 ± 10 years; *P* < 0.001) and had shorter QRS duration (121 ± 33 vs. 126 ± 31 ms; *P* = 0.015) (*Figure 1A*). The proportion of women remained low across implantation periods, while device type varied over time in both sexes (*Figure 1B, C*). Among patients with NYHA III–IV, baseline heart-failure pharmacotherapy was broadly similar between women and men. During 60-month follow-up, appropriate ICD therapy occurred in 21.3% of women and 27.2% of men (*P* = 0.04), ventricular tachycardia in 16.7% and 21.1% (*P* = 0.1), ventricular fibrillation in 4.7% and 6.1% (*P* = 0.4), and all-cause death in 8.7% and 12.4% (*P* = 0.08), respectively. Freedom from appropriate ICD therapy was higher in women, whereas cumulative incidence curves for arrhythmic and non-arrhythmic death were lower (*Figure 1D, E*). In multivariable analysis, male sex was not significantly associated with appropriate ICD therapy [odds ratio (OR) 1.27, 95% confidence interval (CI) 0.94–1.73; *P* = 0.12]. Male sex was associated with higher odds of all-cause death (OR 1.56, 95% CI 1.00–2.44; *P* = 0.05), while older age, lower LVEF, diabetes mellitus, and chronic kidney disease were independently associated with death. No significant interaction for appropriate ICD therapy was observed between sex and implantation period (*P* = 0.32), CRT-D status (*P* = 0.53), or QRS duration (*P* = 0.67). In exploratory sex-stratified models, primary-prevention indication remained associated with lower odds of ICD therapy in both sexes, whereas QRS duration and atrial fibrillation were associated with ICD therapy only in men (*Figure 1F*).

**Figure 1:**
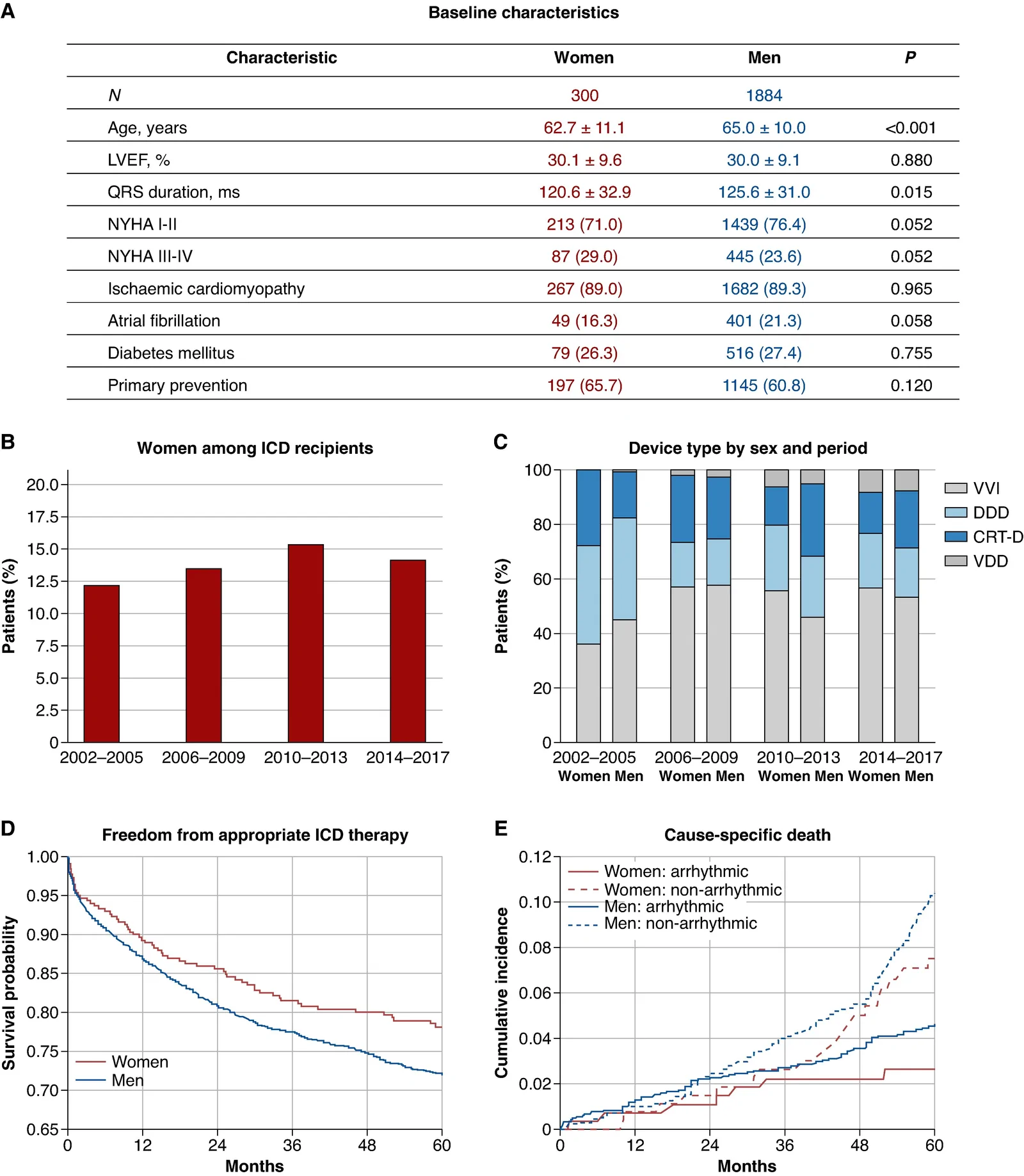

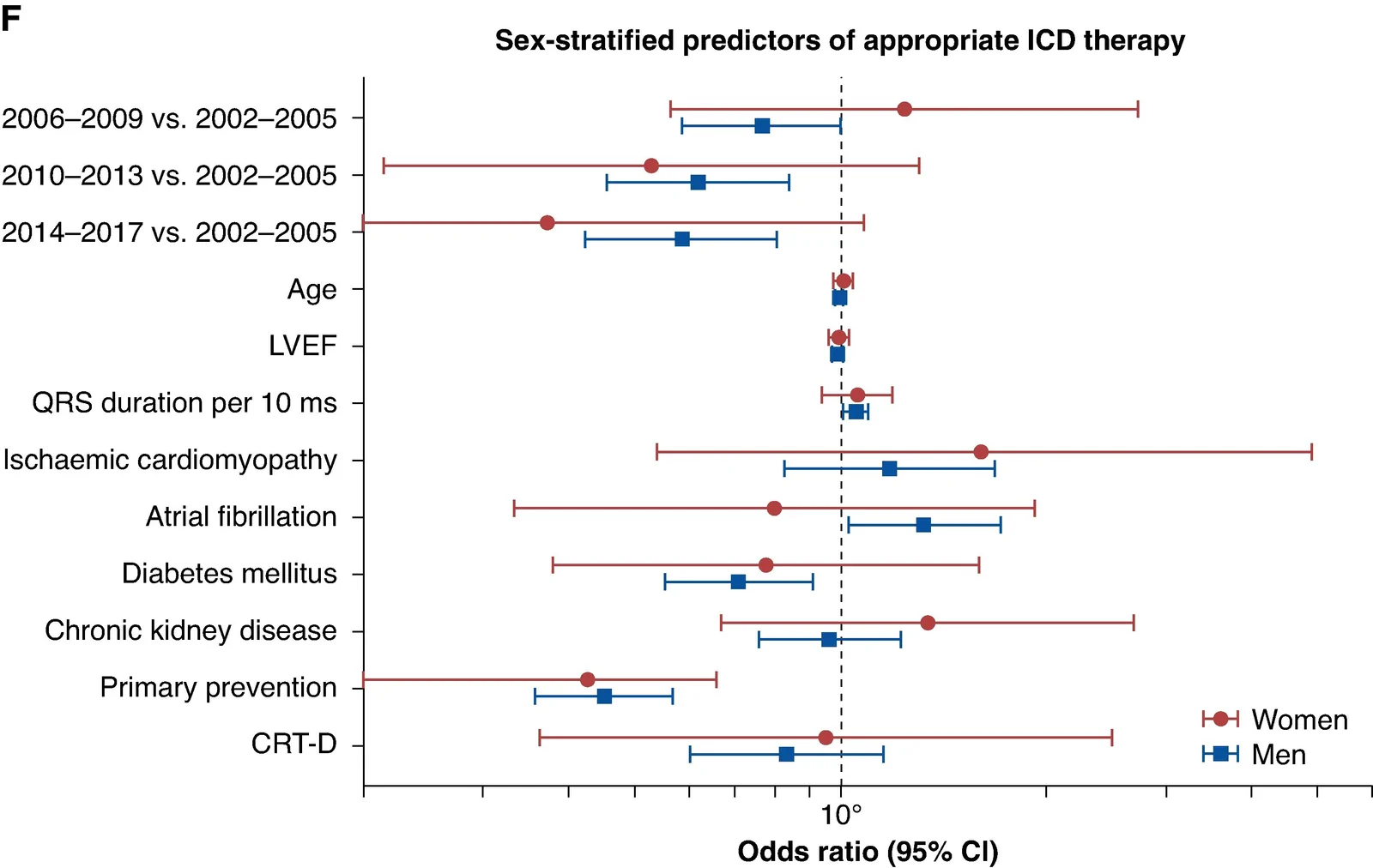
(*A*) Key baseline characteristics by sex. (*B*) The proportion of women among ICD recipients across implantation periods. (*C*) Device-type distribution by sex and period. (*D*) Kaplan–Meier curves for freedom from appropriate ICD therapy. (*E*) Cumulative incidence functions for arrhythmic and non-arrhythmic death. (*F*) Exploratory sex-stratified multivariable logistic regression models for appropriate ICD therapy. Abbreviations: CI, confidence interval; CRT-D, cardiac resynchronization therapy defibrillator; ICD, implantable cardioverter-defibrillator; LVEF, left ventricular ejection fraction; OR, odds ratio.

## Discussion

This study complements previous evidence on sex differences in ICD recipients^1–8^ with real-world data from a large European cohort. Women represented a smaller proportion of ICD recipients than men and showed lower rates of appropriate ICD therapy and ventricular arrhythmias. These findings are consistent with the European multicentre registry by Sticherling et al.^7^ and recent MADIT analyses^1^ showing a lower ventricular tachyarrhythmia burden in women. Our analysis extends these observations by including both primary- and secondary-prevention ICD recipients in a predominantly ischaemic cohort, thereby reflecting a broader real-world ICD population than prior trial-based or primary-prevention cohorts. Moreover, the stratification into four implantation periods provided insight into whether sex-related differences persisted despite temporal changes in heart failure therapy, and ICD practice. After multivariable adjustment, sex association was attenuated and did not reach statistical significance. Differences in comorbidity profile, clinical substrate and prevention indication may explain part of the lower observed arrhythmic burden in women. However, the point estimate remained directionally higher in men (OR 1.27), and given the limited number of women and events, a modest independent sex effect cannot be excluded. No significant interaction was identified between sex and implantation period, CRT-D status, or QRS duration, suggesting that observed differences were not confined to specific periods or device subgroups. Although QRS duration was associated with appropriate ICD therapy in the overall cohort,^9^ its prognostic value did not differ by sex, supporting its role as a general marker of arrhythmic substrate^10^ rather than a sex-specific risk factor. Overall, these findings suggest that observed sex-related differences in ICD outcomes remain evident in contemporary real-world practice but may partly reflect differences in underlying disease substrate and indication profile rather than sex alone.

## Limitations

This retrospective study is subject to residual confounding and unmeasured differences across implantation periods. Heterogeneity in ICD programming could not be fully accounted for and may have influenced treated arrhythmia rates. AF treatment strategy, including cardioversion, catheter ablation, anticoagulation, and longitudinal rate- vs. rhythm-control management, was not systematically captured. Catheter ablation and detailed antiarrhythmic treatment pathways were likewise unavailable. Cause-of-death misclassification depended on available documentation, misclassification of arrhythmic vs. non-arrhythmic death cannot be excluded. The limited number of women and events reduced power for sex-stratified and interaction analyses. The findings should therefore be considered hypothesis-generating. The cohort was predominantly ischaemic, derived from two tertiary European centres, and ended in 2017, before widespread use of angiotensin receptor–neprilysin inhibitors and SGLT2 inhibitors.

## Conclusions

In this 15-year European ICD cohort, women comprised a smaller proportion of ICD recipients than men and showed lower rates of appropriate ICD therapy and ventricular arrhythmias. After adjustment, the association between sex and appropriate ICD therapy was attenuated and did not reach statistical significance. No interaction with implantation period, CRT-D status, or QRS duration was detected. Sex-related differences in arrhythmic risk may therefore partly reflect clinical substrate and patient profile.

## Ethics statement

The study complied with the Declaration of Helsinki and was approved by the local ethics committees (EKNZ 2022–02259). Informed consent was obtained from all participants prior to data collection.

## Data Availability

De-identified data and analysis code are available from the corresponding author upon reasonable request and subject to institutional data-sharing agreements.
